# Acute Colonic Pseudo-Obstruction (Ogilvie’s Syndrome) Associated With Herpes Zoster Infection: A Case Report

**DOI:** 10.7759/cureus.87300

**Published:** 2025-07-04

**Authors:** Irlene Rojas Wong, Diego E Chávez Sáenz, Susana E Ortiz Michel, Alejandro Díaz Hernández, Gabriel I Castro Enríquez

**Affiliations:** 1 General Surgery, Hospital Angeles Metropolitano, Mexico City, MEX; 2 Internal Medicine, Centro Médico Nacional 20 de Noviembre Instituto de Seguridad y Servicios Sociales de los Trabajadores del Estado (ISSSTE), Mexico City, MEX

**Keywords:** acute colonic pseudo-obstruction, herpes zoster, ogilvie syndrome, surgery, visceral neuropathy

## Abstract

Ogilvie’s syndrome is an uncommon but potentially serious condition characterized by colonic dilatation in the absence of mechanical obstruction. Although typically associated with postoperative states, trauma, or pharmacologic agents, its occurrence secondary to herpes zoster is exceedingly rare. In this case report, we present a 78-year-old male with generalized abdominal pain, abdominal distention, obstipation, constipation, and herpetiform rash. The treatment modalities include bowel rest, intravenous fluid therapy, and correction of any existing electrolyte imbalances, followed by the initiation of parenteral nutrition, nasogastric tube, and enemas to reduce the risk of complications. Pharmacologic treatment with antiviral therapy and neostigmine is used in select cases. Surgical management is indicated when both therapies fail or complications exist.

The purpose of this case report is to highlight the importance of considering herpes zoster as a rare but significant cause of acute colonic pseudo-obstruction. Early recognition and prompt initiation of antiviral therapy may lead to rapid clinical improvement and prevent complications.

## Introduction

Acute colonic pseudo-obstruction (ACPO), commonly known as Ogilvie’s syndrome, is a functional disorder characterized by significant colonic dilatation without a mechanical obstruction. First described by Sir William Ogilvie in 1948, this syndrome is most commonly described in hospitalized patients with severe comorbidities, particularly in the postoperative settings, trauma, or in association with spinal anesthesia, electrolyte imbalance, association with varicella-zoster virus (VZV) reactivation, leading to herpes zoster (HZ), is notably uncommon and underrecognized. In addition to immunosuppression, other factors that may contribute to VZV reactivation include advanced age, psychological stress, trauma, and chronic medical conditions such as diabetes [1].

The pathophysiological mechanisms by which VZV induces ACPO remain incompletely understood. Proposed theories include that VZV primarily affects sensory neurons; motor involvement can occur through mechanisms such as direct viral spread, neuritis, intrathecal dissemination, and vascular compromise [2].

The association between HZ and ACPO highlights the need for a high index of suspicion in patients with unexplained colonic dilatation, particularly in the setting of immunosuppression or risk factors for VZV reactivation. Early recognition and prompt initiation of conservative management, with or without antiviral therapy and pharmacologic agents such as neostigmine, can significantly reduce morbidity and prevent complications such as ischemia or perforation [3].

This case report aims to contribute to the limited literature on this rare association, emphasizing the importance of considering HZ in the differential diagnosis of ACPO, especially in patients presenting with unexplained colonic dilatation and risk factors for VZV reactivation.

## Case presentation

**Figure 1:**
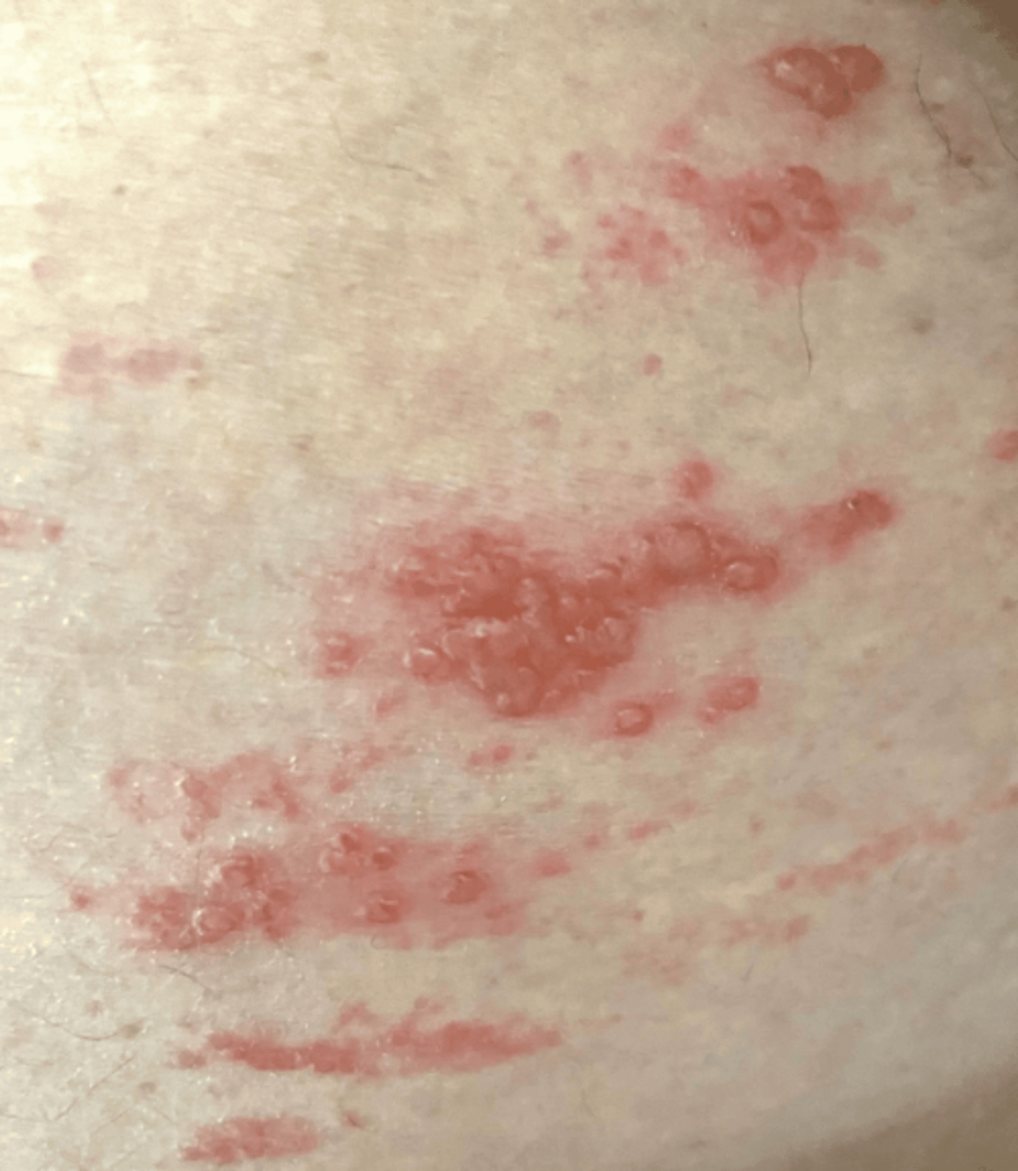
Clinical presentation of herpes zoster dermatosis

A 78-year-old male patient presented to the emergency department with a three-day history of generalized abdominal pain, rated seven out of 10 on the visual analog scale (VAS), associated with abdominal distention, obstipation, and constipation. His medical history included a 12-year history of type 2 diabetes and a one-year history of hypertension, under treatment with glycemic control described as adequate and blood pressure levels controlled. The abdomen was distended and tense, with dermatosis on the left flank characterized by grouped vesicles arranged in a cluster pattern, forming a hemibelt distribution, and tender to palpation (Figure 1). Bowel sounds were hypoactive, and diffuse colonic tenderness was noted without signs of peritoneal irritation. The remainder of the physical examination was unremarkable.

**Figure 2:**
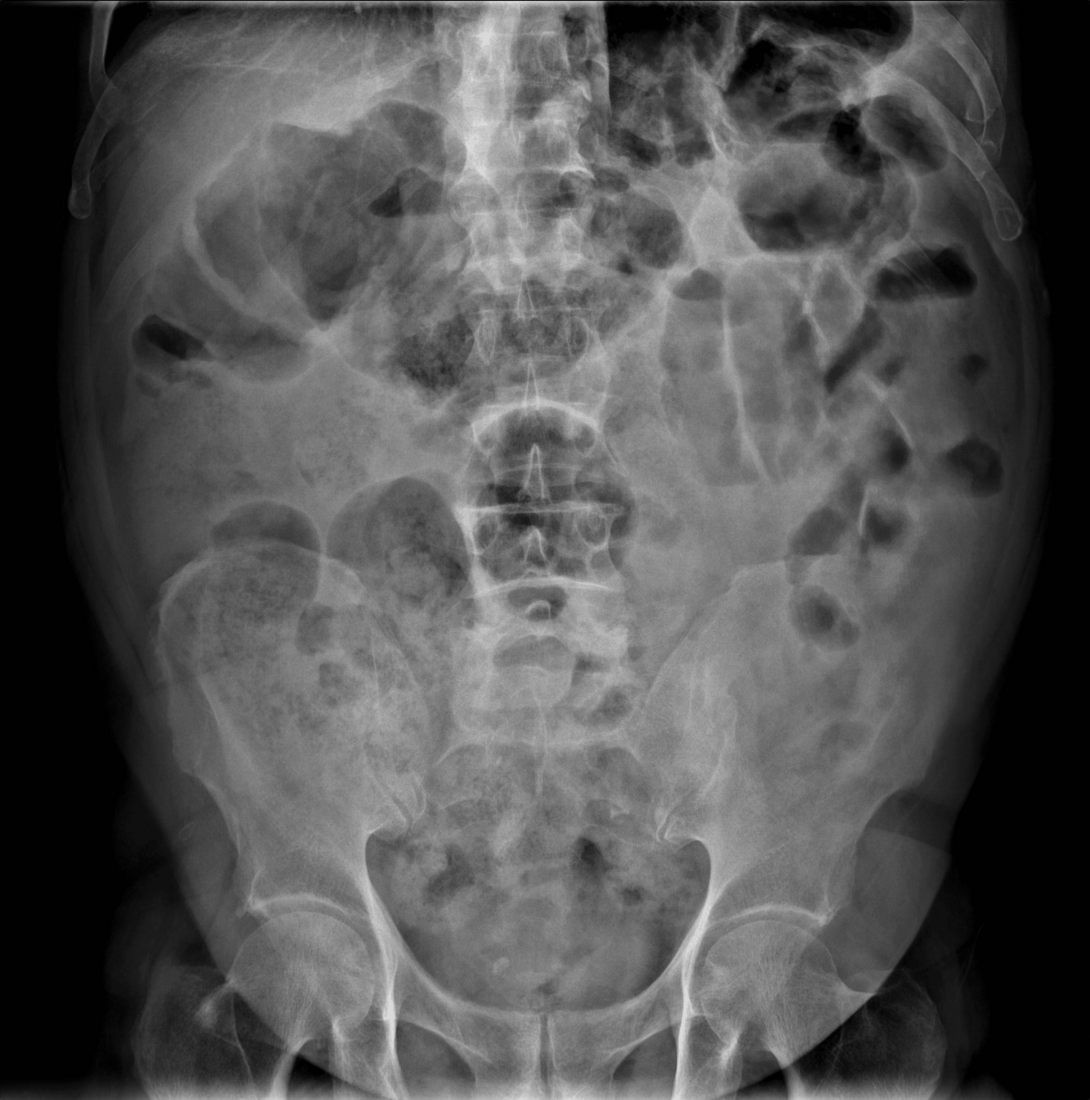
Plain abdominal radiography compatible with functional intestinal obstruction

Laboratory test results show no overwhelming abnormalities, plain abdominal radiography revealed dilated small bowel loops with the presence of multiple air-fluid levels, suggestive of a functional intestinal obstruction (Figure 2).

**Figure 3:**
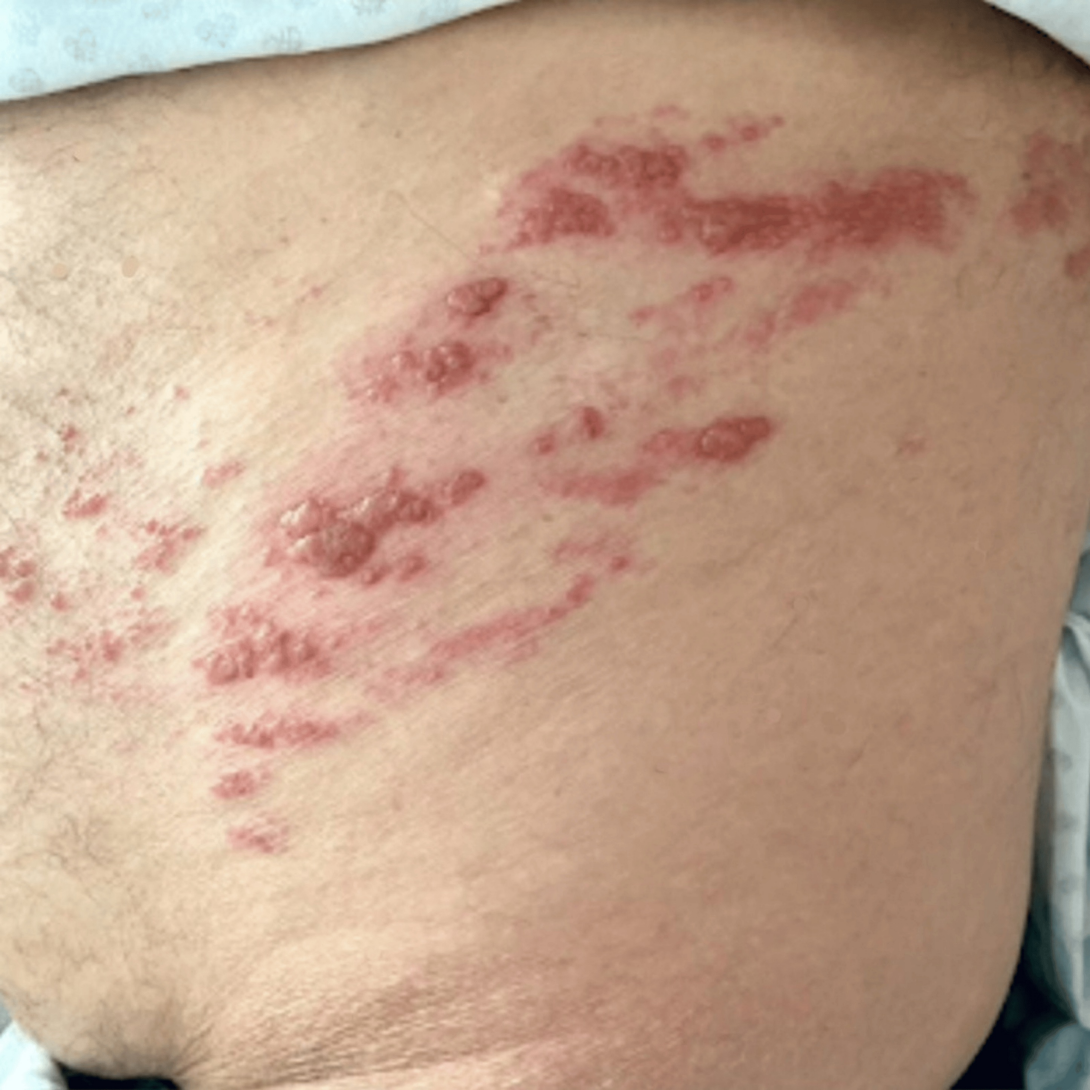
Clinical presentation of the evolution of herpetiform rash involving T-8 to T-9 dermatomes

A non-contrast abdominal computed tomography (CT) scan demonstrated fecal loading throughout the colonic frame, fecalization of the ileum, no identifiable transition point, and findings consistent with a functional intestinal pseudo-obstruction. Two days after, the dermatosis involves T-8 to T-9 dermatomes (Figure 3).

An infectious disease service consult was obtained, which confirmed HZ infection with a high risk of postherpetic neuralgia. The diagnosis of Ogilvie's syndrome secondary to HZ was established. Treatment was started with 1 g of oral valacyclovir for seven days. Clinical improvement was observed within 48 hours of starting medical treatment, presenting bowel movements, tolerating oral intake, and showing marked reduction in abdominal distension.

## Discussion

HZ predominantly affects sensory neurons; in approximately 5% of cases, it involves motor and autonomic fibers, resulting in visceral neuropathies that can manifest as urinary retention or gastrointestinal dysmotility, including ACPO [2].

The mortality rate of ACPO ranges from 7% to 15%; however, in the presence of associated complications, it may increase to as high as 40% [1,3].

The primary clinical symptoms of ACPO are abdominal distention and severe constipation. Other symptoms include abdominal pain, nausea, and vomiting. Laboratory findings are commonly normal. Abdominal X-ray or a CT scan of the abdomen are the main imaging studies used in diagnosis. The sensitivity for detecting colonic distension >9-10 cm in abdominal X-ray is approximately 70-80%. CT scan offers the highest diagnostic accuracy in this setting. CT has a sensitivity of >95% for identifying or excluding mechanical obstruction. It also allows for the assessment of colonic wall integrity, signs of ischemia, perforation, and other potential complications. CT is therefore considered the imaging modality of choice to confirm a diagnosis of ACPO [1].

In association with HZ, a herpetiform rash may appear several days/weeks before or after colonic involvement; in approximately 58% of cases, gastrointestinal symptoms precede the appearance of the characteristic herpetiform rash [3,4]. The dermatomal distribution of the rash commonly involves T8 and T12 (69%); lumbar dermatomes L1-L3 (34%) [5]. Histopathological and immunohistochemical confirmations of ACPO with VZV infection are positive Tzanck and polymerase chain reaction (PCR)-confirmed VZV DNA [3]. 

**Figure 4:**
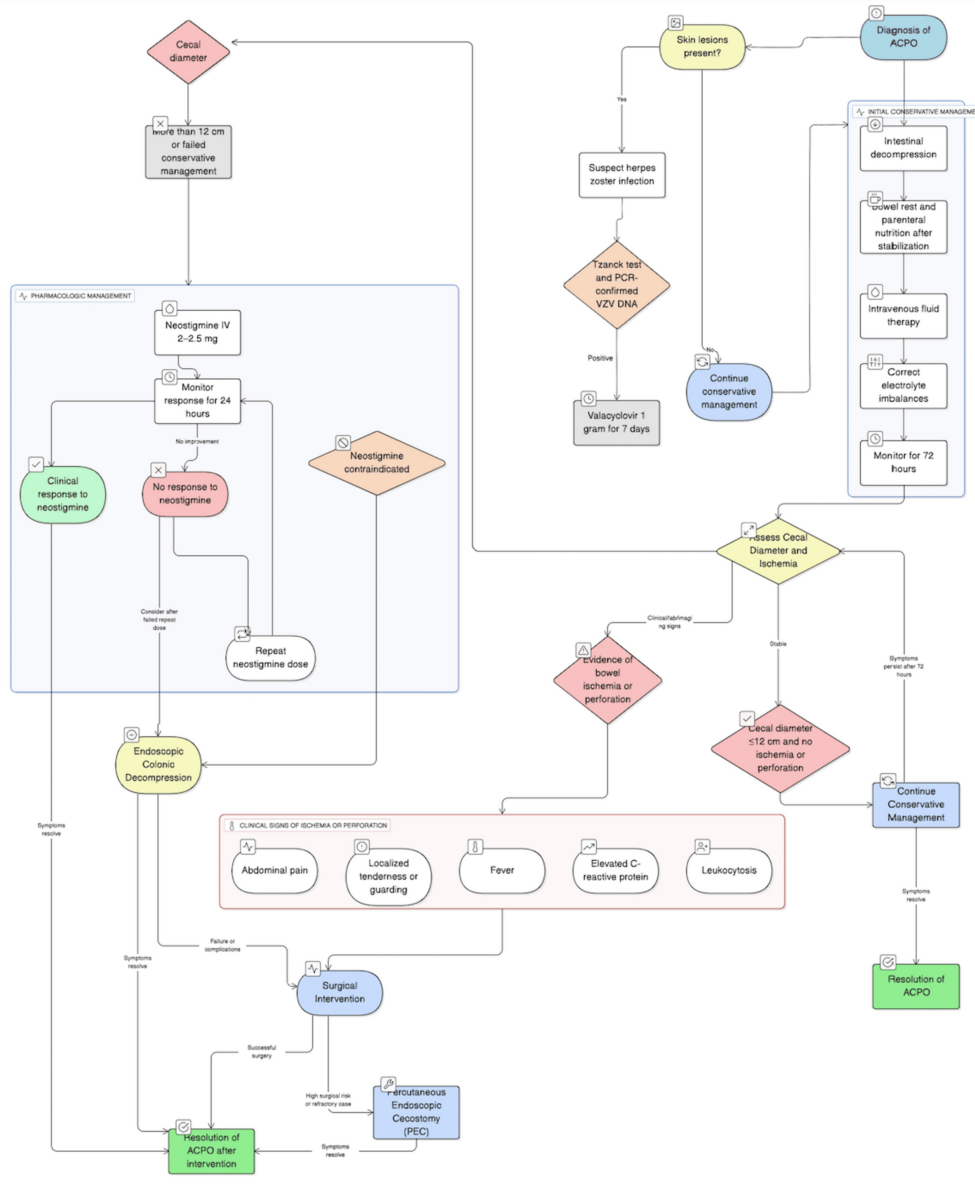
Flowchart of management for Ogilvie’s syndrome and herpes zoster virus infection

The initial management consists of intestinal decompression using a nasogastric tube and enemas to reduce the risk of complications such as perforation (Figure 4). This is complemented by bowel rest, intravenous fluid therapy, and correction of any existing electrolyte imbalances, followed by the initiation of parenteral nutrition this is initiated only after the correction of fluid and electrolyte imbalances to ensure metabolic stability and to minimize the risk of complications such as refeeding syndrome, electrolyte disturbances, and hemodynamic instability. Proper correction of these imbalances is essential to safely support the patient's nutritional needs via parenteral routes [6,7]. Conservative management is continued for 72 hours, provided the cecal diameter is 12 cm and there is no evidence of bowel ischemia or perforation [5].

Pharmacologic treatment with intravenous or oral antiviral therapy, such as acyclovir or valacyclovir, constitutes the main way of management of the skin manifestations, which helps limit viral replication and may contribute to the resolution of associated motor dysfunction and autonomic disturbances [3]. The use of neostigmine (2-2.5 mg) administered intravenously is advised when the cecal diameter surpasses 12 cm or if conservative therapy proves ineffective, as it promotes colonic motility through parasympathetic stimulation. In cases where there is no clinical response within 24 hours of the initial dose, a repeat administration or colonoscopic decompression may be considered. Endoscopic colonic decompression should be considered only in patients in whom neostigmine therapy is contraindicated [1,5,6]. Conservative management is effective in resolving clinical symptoms in approximately 70% of patients diagnosed with ACPO. Pharmacologic intervention with neostigmine has demonstrated efficacy in 60% to 95% of cases, depending on the patient’s clinical context and response to initial dosing [8].

Surgical intervention is reserved for cases complicated by perforation, ischemia, or when both conservative and pharmacologic measures fail to achieve resolution. Clinical indicators suggestive of bowel ischemia or perforation may include abdominal pain, localized tenderness or guarding, signs of systemic infection such as fever, elevated C-reactive protein levels, and leukocytosis [8,9].

In refractory cases of ACPO where conservative and pharmacologic treatments fail or are contraindicated, percutaneous endoscopic cecostomy (PEC) has emerged as a minimally invasive alternative. Vanek et al. presented a case series and comprehensive review supporting PEC as a viable option for decompressing the colon, particularly in patients at high surgical risk, thereby reducing morbidity associated with more invasive procedures [10].

## Conclusions

This case illustrates a rare presentation of Ogilvie’s syndrome triggered by HZ infection, emphasizing the importance of recognizing HZ as an uncommon but clinically relevant etiology of ACPO, particularly in elderly patients with risk factors for VZV reactivation. The association between VZV and colonic dysmotility suggests a neurotropic mechanism disrupting autonomic control of the bowel. Early recognition and prompt diagnosis, supported by clinical examination and imaging studies, enabled early initiation of antiviral therapy, conservative management including bowel rest, supportive care, led to favorable clinical improvement, avoidance of complications, and reduced associated morbidity. A multidisciplinary approach, including infectious disease consultation, is essential for accurate diagnosis and effective management in such atypical presentations. However, in cases where conservative and pharmacologic treatments fail to achieve resolution or complications such as ischemia or perforation arise, surgical intervention remains the definitive therapeutic option.
